# Enhanced Pitch Discrimination for Cochlear Implant Users with a New Haptic Neuroprosthetic

**DOI:** 10.1038/s41598-020-67140-0

**Published:** 2020-06-25

**Authors:** Mark D. Fletcher, Nour Thini, Samuel W. Perry

**Affiliations:** 1 University of Southampton Auditory Implant Service, University of Southampton, University Road, Southampton, SO17 1BJ United Kingdom; 2 Faculty of Engineering and Physical Sciences, University of Southampton, University Road, Southampton, SO17 1BJ United Kingdom

**Keywords:** Sensory processing, Translational research, Human behaviour, Auditory system

## Abstract

The cochlear implant (CI) is the most widely used neuroprosthesis, recovering hearing for more than half a million severely-to-profoundly hearing-impaired people. However, CIs still have significant limitations, with users having severely impaired pitch perception. Pitch is critical to speech understanding (particularly in noise), to separating different sounds in complex acoustic environments, and to music enjoyment. In recent decades, researchers have attempted to overcome shortcomings in CIs by improving implant technology and surgical techniques, but with limited success. In the current study, we take a new approach of providing missing pitch information through haptic stimulation on the forearm, using our new mosaicOne_B device. The mosaicOne_B extracts pitch information in real-time and presents it via 12 motors that are arranged in ascending pitch along the forearm, with each motor representing a different pitch. In normal-hearing subjects listening to CI simulated audio, we showed that participants were able to discriminate pitch differences at a similar performance level to that achieved by normal-hearing listeners. Furthermore, the device was shown to be highly robust to background noise. This enhanced pitch discrimination has the potential to significantly improve music perception, speech recognition, and speech prosody perception in CI users.

## Introduction

Cochlear implants (CIs) are neuroprostheses that allows hundreds of thousands of severely-to-profoundly hearing-impaired people to hear again. To recover auditory perception, an array of micro-electrodes that deliver electrical pulses to the auditory nerve is surgically implanted into the cochlea. Due to anatomical and physical limitations, modern implants use only 12–24 electrodes to transfer sound information to the brain, although only around 8 electrodes are thought to be effective when used together^1,2^. In contrast, in a healthy cochlea, sound information is transferred to the auditory nerve by around 3500 hair cells^3^. Remarkably, despite these limitations, CIs allow the majority of users to identify words in quiet listening environments at an accuracy similar to those with normal hearing^4,5^. However, CI users are typically very poor at detecting pitch changes, which impairs their ability to identify age, sex, and accent^6,7^, as well as perception of speech prosody^8–13^. Speech prosody allows a listener to distinguish statements from questions (e.g. “It's good”. from “It's good?”) and nouns from verbs (e.g. “**Ob**ject” from “Ob**ject**”). It also allows listeners to distinguish emotion (e.g. anger from sadness) and intention (e.g. whether the phrase “nice jumper” was meant as a genuine complement or a sarcastic remark). Impaired pitch discrimination also limits music perception^14^, as pitch conveys crucial melody, harmony, and tonality information. CI users struggle to recognise simple melodies^14–17^ and to discriminate different instruments^14,18,19^ with only around 13% of adult CI users reporting that they enjoy listening to music after implantation^20^.

Traditionally, researchers and manufacturers have attempted to overcome the limitations of CIs by improving implant technology and surgical techniques. However, in recent decades, improvements in CI outcomes have slowed markedly^5,21^. In this study, we take a new approach. Rather than attempting to transfer more pitch information through the implant, we augment the electrical CI signal by delivering pitch information through haptic stimulation on the forearm (“electro-haptic stimulation”^22^). This approach is particularly appealing as this supplementary wearable neuroprosthetic is non-invasive and inexpensive.

The effectiveness of providing sensory information that is usually delivered through one sense using a different sense is well established. Seminal work by Paul Bach-y-rita in the late 1960s showed that, using visual information presented through tactile stimulation on the back, blind people can recognise faces, judge the speed and direction of an object, and complete complex inspection-assembly tasks^23,24^. Later, researchers successfully delivered visual information using sound^25,26^ and basic speech information using haptic stimulation, either on the finger, forearm or wrist^27,28^. More recently, in addition to *substituting* auditory input for haptic input, it has been shown that it is possible to *augment* auditory input with haptic input; three recent studies have shown that CI users’ ability to recognise speech in background noise was enhanced when speech information was presented through haptic stimulation on the wrists^22,29^ or fingertips^30^. Two other recent studies have shown that haptic stimulation can improve melody identification in CI users, using a single-channel haptic stimulation device strapped to the wrist^31^ or fingertip^32^. In the current study, we evaluated the ability of our new mosaicOne_B device, which delivers pitch information through multi-channel haptic stimulation along the forearm, to provide accurate pitch information.

**Figure 1:**
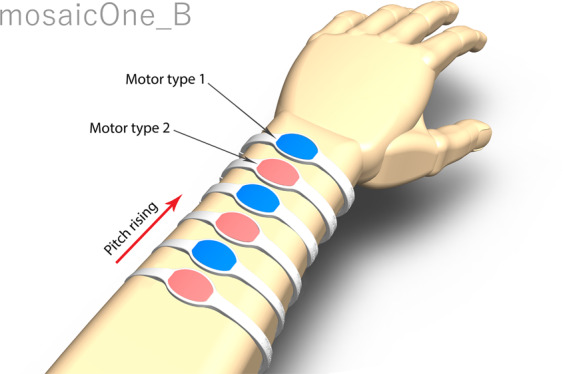
Schematic representation of the mosaicOne_B haptic stimulation device on the forearm. The two interleaved motor types used are represented by different colours.

The mosaicOne_B extracts pitch information from audio in real-time and delivers it through haptic stimulation. The device uses 12 motors, with six along the top and six along the underside of the forearm (see Fig. 1). The motors are activated chromatically, like keys on a piano, with each motor representing a different pitch within a single octave. The mosaicOne_B delivers relative pitch information, meaning that sounds that are exactly an octave apart will produce the same pattern of stimulation. This approach allows for high relative pitch resolution, whilst discarding absolute pitch information (*i*.*e*. information on the pitch of a stimulus within the full scale of perceivable pitches). As even the poorest performing CI users are typically able to discriminate sounds that are an octave apart^33–36^, by using the CI in combination with the mosaicOne_B, CI users are expected to have access to absolute pitch information.

The first aim of this study was to test the limits of pitch discrimination with the mosaicOne_B. Studies using real musical instruments have estimated average pitch discrimination thresholds across CI users of around 80–90% (i.e. 10–11 semitones)^35,36^. Other studies using synthetic sounds (tone complexes) have found average pitch discrimination thresholds of around 10–20% (2–3 semitones)^33,34^. In all of these studies, the variance across subjects was considerable, with some participants only able to discriminate sounds with a pitch difference of slightly less than 100% (one octave) and the best individuals able to discriminate around 3% (0.5 semitones). The performance of the best CI users is similar to pitch discrimination thresholds with musical instruments for normal-hearing listeners^35^. In the current study, we aimed to achieve an average pitch discrimination threshold of 6% (1 semitone) or better. This would allow CI users to track musical melodies (the smallest musical interval for western melodies is typically 1 semitone) and give access to cues for emotion and intention in speech.

Another way in which the mosaicOne_B could aid CI listening is by making it more robust to background noise. CI user performance degrades quickly when there are competing sounds; for example, CI users struggle to discriminate musical instruments when multiple instruments are playing^14,18,19^ or to understand speech in noisy environments^21,22,30^, such as classrooms, busy workplaces, or cafes. A number of studies have shown that, in addition to impairing speech prosody perception, reduced access to information about changes in speech fundamental frequency (*F*_0_; an acoustic correlate of pitch) reduces speech recognition in noise^37,38^. The second aim of this study was to test whether the mosaicOne_B could provide accurate pitch information in the presence of background noise.

Finally, this study aimed to test whether pitch information from different modalities is combined effectively when delivered through audio and haptic stimulation, so that performance with audio and haptic stimulation together is better than with either alone. Alternatively, if one sense gives weak pitch information and the other strong pitch information, the weaker signal may create a distraction that impairs performance. There is a range of anatomical, physiological, and psychophysical evidence to suggest that audio and haptic signals are combined in the brain. Anatomical and physiological studies have revealed extensive connections between auditory and somatosensory neural pathways, from the periphery to the cortex^39–42^. Psychophysical studies have demonstrated both that auditory stimuli can affect haptic perception^43^ and that haptic stimuli can affect auditory perception^44–46^. In one study, it was shown that perception of the dryness of a surface could be modulated by manipulating the accompanying audio^43^. In another set of studies, tactile stimulation was shown to increase perceived loudness and facilitate detection of faint sounds^45,46^. It may therefore be expected that pitch discrimination performance will be better when audio and haptic stimulation are provided concurrently than when either are presented alone.

In the twelve normal-hearing listeners tested in the current study, pitch discrimination was measured with CI-simulated audio alone, haptic stimulation alone, or with audio and haptic stimulation together. The stimuli were harmonic tone complexes that were designed to differ only in pitch (see Methods), so that the results could be generalised to both speech and musical sounds. For each of the three conditions, measurements were made with no background noise, and with background noise at signal-to-noise ratios (SNRs) of either −5 dB or −7.5 dB. These background noise levels were selected as assessment of pitch estimation errors produced by the mosaicOne_B increased at these SNRs (see Methods). It should be noted that these SNRs are far more challenging than those in which CI users are typically able to perform on speech-in-noise recognition tasks^21,22,30^.

## Results

**Figure 2:**
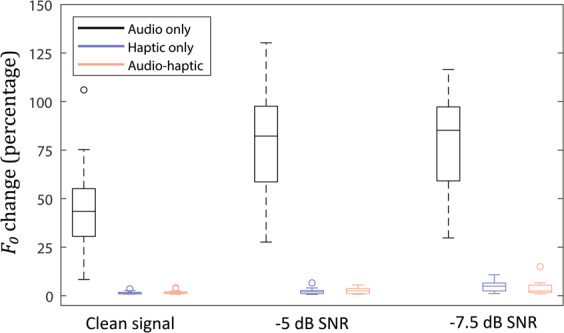
Box plot showing fundamental frequency (*F*_0_) discrimination thresholds across our 12 participants with CI simulated audio only, haptic stimulation only, and audio and haptic stimulation together. Conditions with no background noise and with background noise at either −5 dB or −7.5 dB signal-to-noise ratio (SNR) are shown. The central line on each box shows the median and the bottom and top edges of the box show the 25th and 75th percentiles. The whiskers extend to the most extreme data points that are not considered outliers. Outliers are shown individually as circular symbols.

Figure 2 shows pitch discrimination thresholds with audio stimulation only, with haptic stimulation only, and with audio and haptic stimulation together. Results are shown without background noise and with white background noise at either −5 dB or −7.5 dB SNR. Friedman ANOVAs were conducted with stimulation type (audio only, haptic only, audio-haptic) and noise type (clean, -5 dB SNR, and -7.5 dB SNR) as factors. A significant overall effect of stimulation type was found (χ^2^(2) = 18.17, *p* = <0.001). A significant overall effect of noise was also found for audio (χ^2^(2) = 18.17, *p* = 0.001) and haptic stimulation only (χ^2^(2) = 15.45, *p* = .001). For audio only, pitch discrimination increased from a median change in *F*_0_ of 43.4% without noise (ranging from 8.4% to 106.0% across participants) to 82.2% with noise at −5 dB SNR (ranging from 27.6% to 130%) and to 85.2% with noise at −7.5 dB SNR (ranging from 29.7% to 116.5%). For haptic only, median pitch discrimination was 1.4% without noise (ranging from 0.8% to 3.5%), 2.0% with noise at −5 dB SNR (ranging from 0.6% to 6.6%), and 5.0% with noise at −7.5 dB SNR (ranging from 1.1% to 10.8%). No effect of noise was found for audio-haptic stimulation (χ^2^(2) = 2.09, *p* = 0.35). In the audio-haptic condition, median pitch discrimination thresholds were 1.5% without noise (ranging from 0.8% to 4.1%), 2.5% with noise at −5 dB SNR (ranging from 0.8% to 5.5%), and 2.4% with noise at −7.5 dB SNR (ranging from 0.9% to 15.0%).

Three post-hoc Wilcoxon signed-rank tests (with Holm-Bonferroni correction for multiple comparisons) were conducted to assess the effect of haptic stimulation on pitch discrimination thresholds (see Methods). Pitch discrimination was significantly better with audio-haptic stimulation than with audio alone (*T* = 78, *p* = .001, *d* = 3.76). Median discrimination thresholds improved by 42.0% without noise (ranging from 7.5% to 103.4% across participants), by 80.2% with noise at −5 dB SNR (ranging from 7.5% to 103.6%), and by 80.3% with noise at −7.5 dB SNR (ranging from 5.7% to 95.2%). Pitch discrimination was also significantly better with haptic alone than with audio alone (*T* = 78, *p* = .001, *d* = 3.75). Discrimination improved by 41.9% without noise (ranging from 7.2% to 104.9% across participants), by 79.8% with noise at −5 dB SNR (ranging from 7.0% to 101.0%), and by 80.8% with noise at −7.5 dB SNR (ranging from 7.1% to 91.0%). No difference in pitch discrimination was found between haptic alone and audio-haptic stimulation (*T* = 35, *p* = .791, *d* = −0.05).

## Discussion

In this study, we found that the mosaicOne_B substantially improved pitch discrimination for normal-hearing subjects listening to CI simulated audio. The average pitch discrimination threshold with haptic stimulation was 1.4% (without noise), which corresponds to markedly less than a quartertone, and is comfortably better than our target of 6% (1 semitone). Furthermore, even the worst performer in the current study was substantially better than our targeted average performance across subjects, achieving a pitch discrimination threshold of just 3.5% - comfortably less than a semitone (the minimum pitch change in most western melodies) and similar to pitch discrimination of the best performing CI users^33,34^. For both haptic alone and audio-haptic conditions, some participants achieved pitch discrimination thresholds as low as just 0.8%. This is similar to the performance of normal-hearing listeners for a similar auditory stimulus (although it should be noted that pitch discrimination thresholds for audio is highly sensitive to stimulus parameters)^47^. This enhanced pitch discrimination by the mosaicOne_B has the potential to significantly aid music perception in CI users, as well as speech recognition and speech prosody perception.

The excellent pitch discrimination performance found for the mosaicOne_B was more robust to background noise than may have been expected, with some participants achieving pitch discrimination thresholds of just 0.9% even when the noise was 7.5 dB louder than the signal. In fact, no effect of noise on pitch discrimination thresholds was found. At −7.5 dB SNR (the lowest used in the current study), even the best CI users are unable to perform pitch^48^ or speech recognition tasks^21,22,30^. The absence of an effect of noise was surprising given the greater pitch estimation error by the mosaicOne_B at this low SNR, which led to a wider distribution of motors being activated for a single stimulus (see Methods). It is possible that discrimination was achieved by a comparison of the time-averaged distributions of active motors for each stimulus. A similar process is thought to underlie signal detection in the auditory system^49,50^. The robustness to noise that was achieved by our real-time signal processing strategy is particularly impressive as pitch extraction algorithms tend to be highly susceptible to background noise and can often not be applied in real time^51^. It should be noted that, in the current study, the background noise used was non-harmonic (like environmental sounds such as rain or wind, but unlike competing talkers or background music). Future work is required to explore whether the current approach can also be successfully applied in environments with multiple harmonic sounds.

No difference in performance was found between the audio-haptic and haptic-alone conditions. The absence of a degradation in performance is encouraging, as it indicates that the poor-quality pitch information from auditory stimulation did not distract participants, even after only a small amount of familiarization. It is perhaps not surprising that performance with haptic stimulation was not enhanced by the addition of apparently much poorer pitch information provided through audio stimulation (as indicated by the better performance in the haptic-alone than audio-alone condition). Indeed, it has been observed in several previous studies that the greatest benefit from multisensory integration occurs when senses provide relatively low-quality information when used in isolation^52–54^ (the principal of inverse effectiveness), which was not the case for haptic stimulation. Another reason for the absence of audio-haptic integration may have been the lack of training given. In previous studies, it has been shown that training is critical for audio-haptic integration. This has been shown for haptic enhancement of spatial hearing in CI users^55^ and of speech recognition in noise both for CI users^22^ and for normal-hearing listeners listening to CI simulated audio^29^. It is possible that audio-haptic integration would have been observed in the current study if training was provided.

It should be noted that the current findings do not demonstrate that the auditory percept of pitch was enhanced, but rather that participants were able to access higher-resolution pitch information through haptic stimulation. Participants in a previous study using haptic stimulation to enhance speech-in-noise performance^22^ gave subjective reports that, after training, the speech sounded louder or clearer when haptic stimulation was provided. This indicates that haptic stimulation was able to modulate the audio percept. This idea is supported by psychophysical evidence (discussed in the introduction) that haptic stimulation can modulate auditory perception of loudness and the perception of aspirated and non-aspirated syllables^44–46^. Further work is required to establish whether the auditory percept of pitch can be modulated by haptic stimulation.

The average performance of our participants in the audio-only condition was consistent with previous studies of pitch discrimination in CI users^33–36^. However, previous studies did not use the precise stimulus used in the current study and average performance ranges markedly across studies. In the current study, there was a wide range of performance (of around one octave) across participants. This is also consistent with previous studies with CI users. It should be noted that, while performance of normal-hearing listeners listening to CI simulated audio matched that of CI users, the way that sounds were perceived may have differed.

There are limitations of the current study that should be noted. One such limitation is that pitch discrimination was only demonstrated for a reference signal with an *F*_0_ close to 300 Hz (*F*_0_ was roved). To ensure that the current approach could be applied to signals with different *F*_0_s, mosaicOne_B outputs were assessed for several *F*_0_s (see Methods). The outputs showed good consistency, which indicates that the results of the current study can be generalised to a range of *F*_0_s. A second consideration is the age of the participants who took part (all of whom were under 32 years of age). A substantial portion of the CI user community is significantly older than the population tested in the current study. However, while spatial discrimination on the forearm is known to decline with age, the ability to distinguish between two stimulation points on the forearm remains less than the motor spacing for the mosaicOne_B (3 cm), even in older people^56^. Therefore, the findings of the current study are expected to be translatable to older populations. Finally, only a small amount of training was given in the current study, which may have led to pitch discrimination thresholds being underestimated. Previously, researchers have reported that, for normal-hearing listeners, auditory frequency discrimination performance continues to improve for around two weeks when two hours of training are given each day^57^. Furthermore, studies of enhancement of speech-in-noise performance with haptic stimulation for CI users have shown the importance of training for maximizing benefit^22,29^. Future work should assess whether training can lead to further enhancements in pitch discrimination with the mosaicOne_B.

Several steps are required to maximize the potential of the mosaicOne_B to bring real-world benefits to CI users. Firstly, it will be important to verify the findings of the current study in CI users. Future work should also seek to optimize the pitch extraction techniques used to reduce estimation errors in noise. Another important step, already discussed, is to assess the ability of the mosaicOne_B to effectively extract pitch cues in the presence of multiple harmonic sounds. Additionally, future studies should assess the effectiveness of the mosaicOne_B for improving speech perception, both in quiet and in noise, and music perception. Future developments to the mosaicOne_B could also include the exploitation of spatial hearing cues. CI users have poor access to spatial cues and are extremely poor at locating sounds^58^, which can lead to impaired threat detection and sound source segregation. For example, it is well established that access to spatial hearing cues can enhance detection of signals, such as speech, in noise^59,60^. A recent study in CI users has shown strong evidence that haptic stimulation can be used to enhance localisation of sounds^55^ and a similar approach might be implemented on the mosaicOne_B. Finally, a wearable neuroprosethic like the mosaicOne_B could include additonal aids to everyday activities. It could incorporate features such as a wake-up alarm (as CI users typically charge their implants during the night), and could link to smart devices in the Internet of Things, such as telephones, doorbells, baby monitors, ovens, and fire alarms.

The results of the current study demonstrate that the mosaicOne_B can extract and deliver precise pitch information through haptic stimulation. The device has been shown to be remarkably robust to non-harmonic background noise, which is common in real-world environments. The mosaicOne_B has several properties that make it suitable for a real-world application: stimulation was delivered to the forearm (a suitable site for a real-world use), the signal processing was performed in real-time, and the haptic signal was delivered using lightweight, low-powered, compact motors. The findings of the current study suggest that the mosaicOne_B could offer a non-invasive and inexpensive means to improve speech and music perception in CI users.

## Methods

### Participants

Twelve participants (3 male and 9 female, aged between 22 and 31 years old) were recruited from the staff and students of the University of Southampton, and from acquaintances of the researchers. Participants gave written informed consent and no payment was given for participation. All participants reported no hearing or touch issues, had received no musical training, and did not speak a tonal language. Vibrotactile detection thresholds were measured at the fingertips of the left and right index fingers. Thresholds were measured at 31.5 Hz and 125 Hz, following conditions and criteria specified in ISO 13091-1:2001^61^ (the fingertip was used as there are no published standards for normal wrist or forearm sensitivity). All participants had vibrotactile detection thresholds within the normal range (<0.4 ms^−2^ RMS at 31.5 Hz, and <0.7 ms^−2^ RMS at 125 Hz^61^), indicating no touch perception issues. Participants were also assessed by otoscopy and pure-tone audiometry. Participants had hearing thresholds not exceeding 20 dB hearing level (HL) at any of the standard audiometric frequencies between 0.25 and 8 kHz in either ear.

### Stimuli

In testing and task familiarisation (see Procedure), the reference stimulus was a harmonic complex, with an average *F*_0_ of 300 Hz (within the range of *F*_0_s found for many musical instruments, approximately central to the range of *F*_0_s found in human speech^62^, and the frequency at which pitch cues are reduced for CI users^14^). The *F*_0_ was roved by ±5% on each presentation (with a uniform distribution). The stimulus comprised of equal-amplitude harmonics generated up to 24 kHz (the Nyquist frequency). This signal was band-pass filtered between 1 kHz and 4 kHz, with 12^th^ order (72 dB per octave) 0-phase Butterworth filters, to remove non-pitch cues (such as differences in the brightness of the sound^63^, as discussed in Mehta and Oxenham^64^]). The signal had a duration of 500 ms, with 20 ms quarter-sine and -cosine onset and offset ramps. The target and reference stimuli were separated by 300 ms. The target and reference stimuli were the same, except that the *F*_0_ of the target stimuli was adjusted following the adaptive track described in the procedure section. The level of the target and reference was nominally set to 65 dB SPL (RMS), but was roved on each presentation within a ±3 dB range (with a uniform distribution) to reduce potential loudness cues. The masking stimulus was a white noise, selected to equally mask each of the components of the harmonic complex.

The audio stimuli were processed using the SPIRAL vocoder to simulate CI listening. The SPIRAL vocoder is an advanced CI simulator that aims to bridge the gap between traditional tone- and noise-based simulations^65^. The SPIRAL was set to simulate 22 CI electrodes (with a current decay slope of 16 dB per octave) using 80 carrier tones. The test stimuli were delivered to the participants’ right ear only. In the audio alone and audio-haptic conditions, pink noise at a level of 55 dB SPL was delivered to the left ear to mask any audio cues from the mosaicOne_B. In the haptic-alone condition, the pink noise was delivered to both ears.

In the mosaicOne_B familiarization app (see Procedure), two stimulus types were used. In both of the app’s modules, CI simulation was not applied to the audio. In the pitch slider module, a constant tone was presented, and the frequency was adjusted between D3 and B3 on the chromatic scale based on the slider position. In the interval training module, two tones were presented. The tones were 500 ms long, with 20 ms quarter-sine and -cosine onset and offset ramps and were separated by a 100 ms gap. Frequencies were selected at random between D3 and B3 on the chromatic scale.

### Tactile signal processing

**Figure 3:**
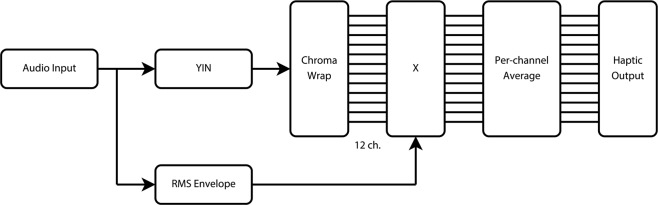
Schematic illustration of the signal processing chain for haptic signal generation.

Melody in music and prosody in speech are typically conveyed in sub-octave frequency shifts, with the absolute height of the pitch being largely irrelevant. In the current study, we used a pitch chroma analysis, which groups frequencies by octave to produce a spectral representation of relative pitch, discarding absolute pitch height information. A schematic representation of the signal processing chain that was used to convert audio to a tactile signal is illustrated in Fig. 3. The haptic signal was generated by first estimating the *F*_0_ and amplitude envelope of the input signal. *F*_0_ was estimated using YIN, implemented in the Max Sound Box toolbox (version 2018-3, IRCAM, Paris, FR). A 14 ms window size was used (giving a minimum possible *F*_0_ estimation of approximately 70 Hz) with no downsampling. The resulting *F*_0_ estimate was then used to activate one of the 12 shakers on the mosaicOne_B. This was achieved by first mapping the *F*_0_ to the MIDI scale, a commonly used scale for relating musical pitch to frequency. This representation was then assigned to one of 12 frequency channels. The full frequency mapping was defined as:

$${f}_{wrap}[n]=\,\mathrm{mod}\left(69+12\cdot {\log }_{2}\left(\frac{{F}_{0}[n]}{440}\right),12\right),$$

$${y}_{i}[n]=\{\frac{1,\,i={f}_{wrap}[n]}{0,\,otherwise},$$

where *f*_*wrap*_ is an integer in the range $0\le {f}_{{\rm{wrap}}} < 12$, and *y*_*i*_ is the channel at index *i*.

**Figure 4:**
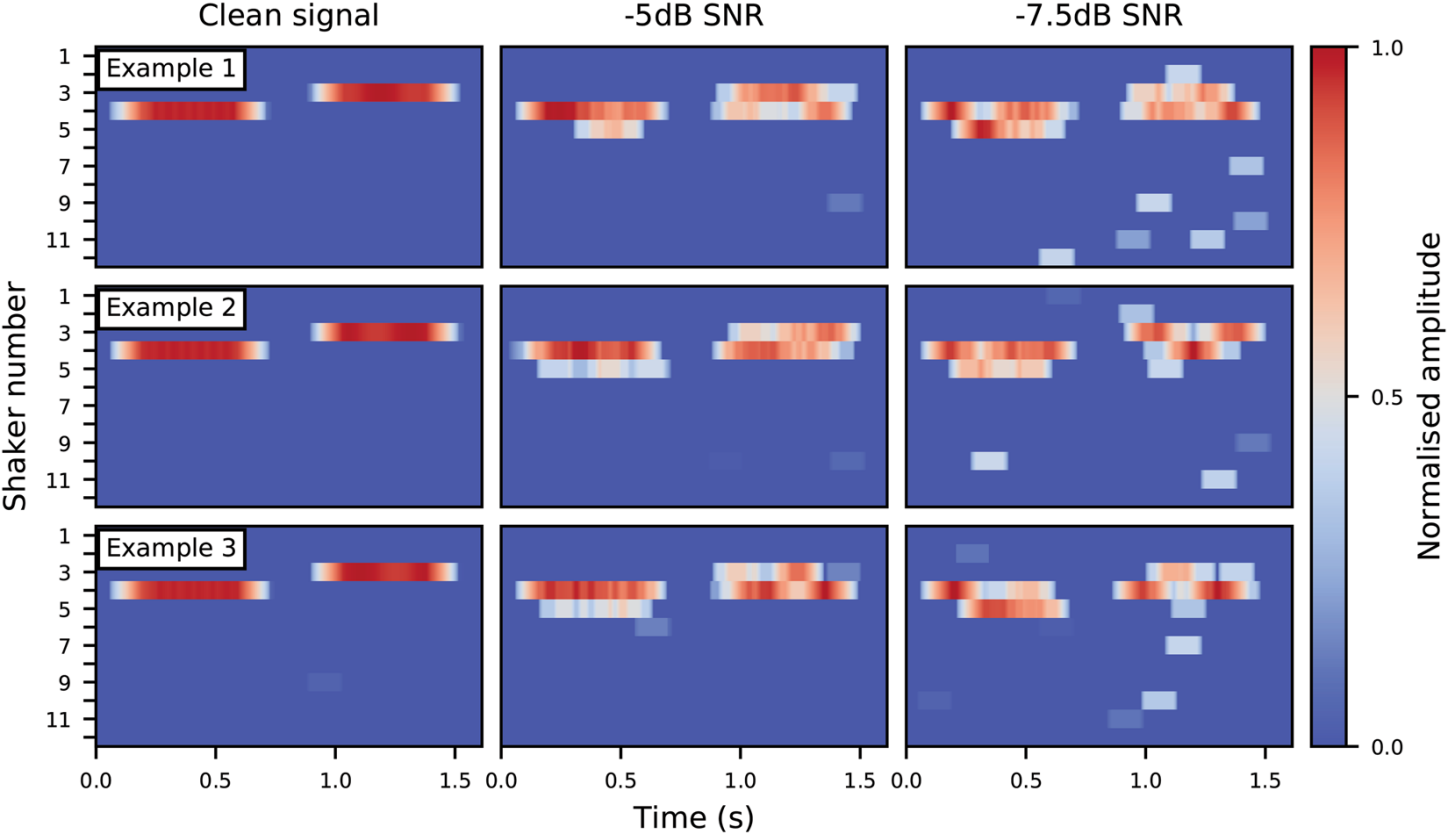
Haptic signals in response to three example harmonic complexes for the clean, −5 dB and −7.5 dB SNR conditions. The reference signal is followed by a target signal with an *F*_0_ increased by 5%.

The RMS amplitude envelope of the input audio was calculated in parallel, using a 14 ms window. The activated channel was then multiplied by this envelope. Finally, a moving RMS average of each of the 12 channels was calculated using a 125 ms window. This per-channel averaging acted as a simple noise-reduction method. This helped reduce the effects of artefacts produced by the *F*_0_ estimation algorithm as background noise increased. The haptic output in response to the harmonic complexes (described in the Stimuli section) is illustrated in Fig. 4.

**Figure 5:**
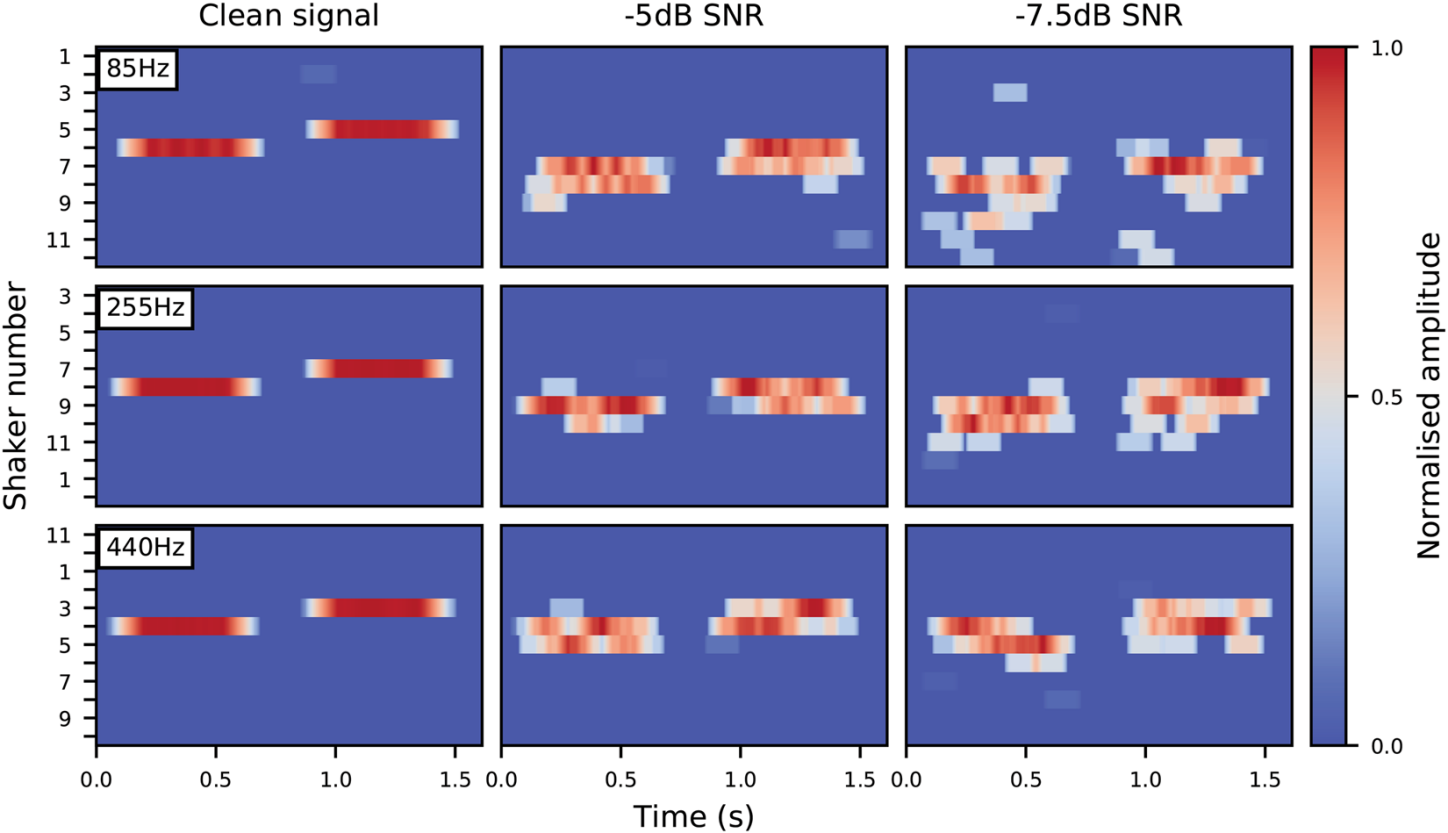
Haptic signals in response to a harmonic complex (sawtooth wave) at 85 Hz, 255 Hz and 440 Hz in clean, −5dB and −7.5 dB SNR conditions. The reference signal is followed by a target signal with an *F*_0_ increased by 5%. Shaker numbers are offset to centre the reference stimulus for display purposes.

The performance of the algorithm was assessed for different stimulus frequencies. Sawtooth waves at 85 Hz (lowest average *F*_0_ of typical male speech^62^), 255 Hz (highest average *F*_0_ of typical female speech^62^) and 440 Hz (standard tuning pitch for western music). A sawtooth harmonic complex was used as it consists of equivalent odd and even harmonics (that decrease in amplitude with increasing frequency – as is typical of real-world stimuli, such as speech). Bandpass filtering was not applied as removal of non-pitch cues was not necessary. Figure 5 illustrates the algorithm’s performance. Performance at decreasing SNRs is comparable to the test stimulus, with marginally poorer performance at 85 Hz for the −7.5 dB SNR condition. Additionally, for the −5 and −7.5 dB SNR conditions at 85 Hz and 255 Hz, estimates are offset by 1–2 shakers relative to the clean condition. These errors are due to the inaccuracy of initial *F*_0_ estimation, and the non-linear mapping of frequency (which requires greater precision of *F*_0_ estimation at lower frequencies). Despite these errors, relative pitch differences appear largely unaffected.

### Apparatus

During pure-tone audiometry, participants were seated in a sound-attenuated booth with a background noise level conforming to British Society of Audiology recommendations^66^. Audiometric measurements were conducted using a Grason-Stadler GSI 61 Clinical Audiometer and Telephonics 296 D200-2 headphones. Vibro-tactile threshold measurements were made using a HVLab Vibro-tactile Perception Meter with a 6-mm contactor that had a rigid surround and a constant upward force of 2 N (following International Organization for Standardization specifications^61^). This system was calibrated using a Bruel & Kjaer (B&K) calibration exciter (Type 4294).

The experiment took place in a quiet listening room. During testing, the experimenter sat behind a screen with no line of site to the participant. The participants responded by pressing buttons on a iiyama ProLite T2454MSC-B1AG 24-inch touchscreen monitor. All stimuli were generated using custom MATLAB scripts (version R2019a, The MathWorks Inc., Natick, MA, USA) and controlled using Max 8 (version 8.0.8). Both audio and haptic signals were played out at a sample rate of 48 kHz via a MOTU 24Ao soundcard (MOTU, Cambridge, MA, USA). Audio was presented using ER-2 insert earphones (Etymotic, IL, USA) and the haptic signal was delivered through the mosaicOne_B via the mosaicOne_B haptic interface (for amplification of haptic signals). Audio stimuli were calibrated using a B&K G4 sound level meter, with a B&K 4157 occluded ear coupler (Royston, Hertfordshire, UK). Sound level meter calibration checks were carried out using a B&K Type 4231 sound calibrator.

Haptic stimulation was delivered using the mosaicOne_B (Fig. 1 shows a schematic representation of the device). The mosaicOne_B had twelve motors, with six strapped to the top and six to the bottom of the forearm. The motors were attached using six elastic straps, fastened with Velcro. Two motor types, the Precision Microdrives 304–116 5-mm vibration motor (labelled “Motor type 1” in Fig. 1) and the Precision Microdrives 306-10H 7-mm vibration motor (labelled “Motor type 2” in Fig. 1) were used in an interleaved fashion, with each motor separated by 3 cm. The bottom motors were arranged in reverse order to maximize the distance between motor types. The motors were calibrated so that the driving signal extrema corresponded to the output amplitude extrema (maximum amplitudes of 1 and 1.84 G, respectively). This maximised the dynamic range of the motors. The different motor types have different operating frequencies of 280 Hz for the 5 mm motor and 230 Hz for the 7 mm motor. This configuration was selected to maximize differentiation between motors by allowing the user to exploit both skin location and stimulation frequency cues. The different motors were expected to be discriminable in frequency based on frequency discrimination thresholds for vibrotactile stimulation^67^. The motors were also expected to be spatially discriminable, even in older users, based on two-point discrimination thresholds^56^. Note that it has been argued that two-point discrimination thresholds likely over-estimate the minimum location separation required to discriminate motors^24^. This suggestion was supported by informal testing during development of the mosaicOne_B.

### Procedure

The experiment had three phases, all of which were completed in a single session lasting around two hours. The first phase was the screening phase. During screening, participants first completed a questionnaire to ensure that they (1) had no conditions or injuries that may affect their touch perception, (2) had not been exposed to sustained periods of intense hand or arm vibration at any time, (3) had no recent exposure to hand or arm vibration, (4) had no conditions or injuries that may affect their hearing perception, (5) had received no musical training at any time, or (6) did not speak a tonal language. Next, audiometric hearing thresholds were measured to ensure participants had normal hearing (thresholds <20 dB HL). Thresholds were measured following British Society of Audiology guidelines^66^. Following this, vibrotactile detection thresholds were measured at the fingertip, to check for normal touch perception (<0.4 ms^−2^ RMS at 31.5 Hz, and <0.7 ms^−2^ RMS at 125 Hz^61^). Thresholds were measured following the protocol recommended by the International Organization for Standardization^61^. Finally, otoscopy was performed to ensure insert earphones could safely be used. If the participant passed all screening stages, they continued to the familiarization phase.

In the familiarization phase, participants first used an app developed to familiarize them with the mosaicOne_B. Participants used the app for 5–10 minutes and were invited to ask questions if anything was unclear. The app consisted of a pitch slider and an interval training module. For each module, participants could switch between haptic only, audio-haptic and audio only modes. In both modules, CI simulation was not applied to the audio. In the pitch slider module, a constant tone was played, and the frequency of the tone was adjusted based on slider position. In the interval training module, participants could select either a “Low → High” or “High → Low” button, which determined the pitches of two consecutive tones. The number of presentations was not limited, but any given presentation could not be repeated.

After using the app to familiarize themselves with the device, participants were familiarized with the task used in the testing phase. Participants completed a short practice session of 15 trials for each condition. In the testing and task familiarization, a two-alternative forced-choice task was used in which participants were asked to judge which interval contained the sound or vibration stimulus with the higher pitch. Participants used two buttons labelled “1” and “2” to select whether the first or second stimulus was higher in pitch. Visual feedback was given, indicating whether the response was correct or incorrect. The pitch difference between intervals was initially set at +80% of the reference pitch and was then varied using a one-up, two-down adaptive procedure, with percentage difference varying by 10% for the first two reversals, 5% for the third reversal, and 1% for the remaining four reversals. Thresholds for each track were calculated as the mean of the last four reversals. The order of conditions (audio only, audio-haptic, and haptic only) was counterbalanced across participants, and the noise conditions (no noise, noise at −5 dB SNR, and noise at −7.5 dB SNR) were presented in a random order for each condition.

The experimental protocol was approved by the University of Southampton Faculty of Engineering and Physical Sciences Ethics Committee (ERGO ID: 47769). All research was performed in accordance with the relevant guidelines and regulations.

### Statistics

The data were analysed using Friedman ANOVAs (with Bonferoni-Holm correction for multiple comparisons). Three planned post-hoc Wilcoxon signed-rank tests were also performed (also Bonferoni-Holm corrected). Non-parametric tests were used as the data was not normally distributed.

## Data Availability

The dataset from the current study is publicly available through the University of Southampton’s Research Data Management Repository at: 10.5258/SOTON/D1401.
